# Socioeconomic and Healthcare Indicators and Colorectal Cancer Burden: Analysis of Eurostat and Global Burden of Disease Study 2021 Data

**DOI:** 10.3390/cancers17132075

**Published:** 2025-06-21

**Authors:** Nóra Kovács, Orsolya Varga

**Affiliations:** 1 Department of Public Health and Epidemiology, Faculty of Medicine, University of Debrecen, 4028 Debrecen, Hungary; varga.orsolya@med.unideb.hu; 2 Syreon Research Institute, 1142 Budapest, Hungary

**Keywords:** healthcare indicators, disease burden, colorectal cancer, inequality, Gini index

## Abstract

**Background/Objectives:** Colorectal cancer (CRC) is a leading cause of cancer-related morbidity and mortality worldwide, posing a substantial health and economic burden. Despite advances in screening and treatment, significant socioeconomic and healthcare-related disparities persist across European Union (EU) member states. This study aims to identify trends and inequality in CRC burden over time and to explore the relationship between country-level socioeconomic and healthcare indicators and CRC burden across EU member states. **Methods:** Age-standardized mortality, years lived with disability (YLD), years of life lost (YLL), and disability-adjusted life years (DALY) rates were extracted from Global Burden of Diseases Study 2021 for 24 EU countries. Socioeconomic and healthcare indicators were extracted from Eurostat between 2005 and 2021. The Gini index was calculated to evaluate CRC-related health inequality, and generalized linear mixed models were used to assess the link between indicators and disease burden. **Results:** The Gini index for age-standardized YLDs declined from 0.19 to 0.12 between 1990 and 2021, while inequality in YLL (from 0.11 to 0.16), DALY (from 0.11 to 0.15), and mortality rates (from 0.12 to 0.14) increased. The number of practicing physicians (*p* < 0.05) and higher levels of education (*p* < 0.001) were related to lower death, DALY, YLD, and YLL rates. Conversely, greater income inequality was linked to higher mortality, DALY, and YLL rates (*p* < 0.05). **Conclusions:** Our findings underscore that, in addition to expanding organized screening programs, enhancing physician availability and addressing socioeconomic inequalities are essential for reducing the burden of CRC.

## Simple Summary

Colorectal cancer (CRC) remains a major health and economic challenge across the European Union (EU). This study aimed to examine trends and inequalities in CRC burden and to explore its association with country-level socioeconomic and healthcare indicators across 24 EU member states. CRC burden was measured using age-standardized mortality, years lived with disability (YLD), years of life lost (YLL), and disability-adjusted life years (DALY) rates, which were obtained from the Global Burden of Disease Study 2021. Socioeconomic and healthcare data were collected from Eurostat between 2005 and 2021. While inequality in YLD rate decreased, disparities in mortality, YLL, and DALY rates increased. Higher numbers of physicians and higher education levels were linked to lower CRC burden, while a greater income inequality was linked to higher burden. These findings emphasize the importance of expanding screening programs, improving healthcare capacity, and reducing social inequalities to address CRC disparities in the EU.

## 1. Introduction

Colorectal cancer (CRC) is a major global public health concern, ranking as the third most common cancer worldwide. In 2020, there were over 1.9 million new cases and approximately 930,000 deaths attributed to CRC, highlighting its substantial contribution to global morbidity and mortality [1,2].

Within the European Union (EU), it accounted for 11.6% of all cancer deaths in 2021 [3]. According to the Global Burden of Disease Study 2021, CRC was responsible for more than 175,000 deaths and over 3.3 million disability-adjusted life years (DALYs) across EU member states, with considerable variation across countries [2,4].

In 2018, the economic burden of colorectal cancer in the European Union was estimated at €19 billion (€12.2 billion for colon and €6.8 billion for rectum cancers), covering direct healthcare, informal care, and indirect costs. Informal care and indirect costs combined were closely equal to direct costs, demonstrating the broader social impact of the disease [5].

CRC disproportionately impacts individuals from low socioeconomic backgrounds and certain racial minorities, partly due to greater exposure to risk factors like poor diet, inactivity, and reduced access to preventive measures such as chemoprevention [6]. Screening programs have contributed substantially to the reduction in CRC incidence and mortality rates across many European countries over the past two decades, particularly in countries where long-standing screening programs are in place (e.g., Austria, the Czech Republic, and Germany) [7]. However, participation remains suboptimal, and significant socioeconomic inequalities persist. Numerous studies have shown that individuals from lower social groups are considerably less likely to participate in CRC screening [8,9]. These disparities, however, are not limited to screening but extend to treatment access, survival, and overall mortality. Previous studies found that patients with low SES are more likely to experience delays in treatment [10] and are less likely to receive surgery and adjuvant therapies [11], which in turn negatively impacts prognosis and survival [11,12,13].

Healthcare system capacity is also a key determinant of colorectal cancer burden in terms of access to screening, early diagnosis, availability of treatment, and follow-up care. A recent meta-analysis found that regions with limited healthcare resources (including a shortage of medical specialists) experience longer diagnostic intervals [14]. All of these factors are critical for improving patient outcomes and reducing mortality [15,16,17].

While individual-level CRC disparities are well documented, comparative EU-wide studies on CRC burden remain limited. To address this gap, the present study aimed to explore how selected socioeconomic and healthcare indicators are related to CRC burden across EU member states by linking data from the Global Burden of Disease Study (GBD) 2021 with publicly available datasets from the Statistical Office of the European Union (Eurostat).

## 2. Materials and Methods

### 2.1. Study Design and Data Sources

Data for this study were obtained from two publicly available databases. Age-standardized disease burden data for colon and rectum cancer were retrieved from the Global Health Data Exchange (GHDx), while healthcare and socioeconomic indicators for EU member states were sourced from the Eurostat database. Data were obtained for 24 of the 27 EU member states. Greece, Portugal, and Slovakia were excluded because data on the number of practicing physicians were not available for these countries during the study period.

The Global Burden of Disease (GBD) 2021 study provides comprehensive estimates for 371 diseases and injuries, 88 risk factors by sex and age across 204 countries and territories [18]. GBD 2021 provides annual estimates between 1990 and 2021 available through the publicly accessible GHDx platform (http://ghdx.healthdata.org/gbd-results-tool (accessed on 10 May 2025)).

Data from 24 EU member states were collected using the GBD 2021 database. We utilized age-standardized rates of death, disability-adjusted life years (DALYs), years lived with disability (YLDs), and years of life lost (YLLs) associated with colon and rectum cancer at European Union and national levels. All rates were expressed per 100,000 population. Age-standardized rates were used to ensure comparability over time and between populations, given the demographic shifts in population growth and aging. YLL represents the years of life lost due to a specific cause when compared to the standard life expectancy. YLD refers to the years lived with any disability, weighted by the severity of the health condition. The DALY was used to measure the total burden of chronic diseases by summing YLD and YLL values [19,20]. The detailed methodology for calculating age-standardized rates and DALYs can be found in GBD publications [21].

The Eurostat database provides statistics for European Union member states using various data sources, including the European Union Statistics on Income and Living Conditions (EU-SILC), which has provided annually updated and comparable cross-country data on income, poverty, social exclusion, and living conditions since 2003. Relevant indicators related to healthcare and socioeconomic status were selected for analysis. The following indicators were extracted from the Eurostat database for 24 EU member states, covering the period 2005–2021 based on data availability: healthcare indicators included current healthcare expenditure in percentage of gross domestic product (GDP), number of hospital beds, and practicing physicians; socioeconomic indicators included income inequality, unemployment rate, and proportion of population with tertiary education. The definitions of each indicator are available in Supplementary File.

### 2.2. Statistical Analysis

The data on Eurostat indicators were available for limited period, and the trend analysis of country-specific age-standardized rates was conducted using the Joinpoint regression analysis during 2005–2021. The average annual percent change (AAPC) was calculated with log-transformation and automatic selection of the best fitting model. All Joinpoint analyses were performed using Joinpoint Regression Program (Version 5.4.0—16 April 2025, Statistical Methodology and Applications Branch, Surveillance Research Program, National Cancer Institute) [22].

We used the Gini index to assess the CRC-associated health inequality across 24 member states over time from 1990 to 2021. The Gini index is a measure of inequality, calculated based on the Lorenz curve. The Lorenz curve shows the cumulative proportion of the population ranked by health, as well as a cumulative proportion of health variable within the population. A hypothetical diagonal line at 45° is drawn to represent a perfect distribution of health within the population. The Gini coefficient measures deviation from an equal distribution and is calculated based on the area between the 45° line and the Lorenz curve. This coefficient ranges from 0 (perfect equality) to 1 (total inequality), with a higher value indicating greater inequality [23,24]. In this study, the health measures used to calculate the Gini index were the annual age-standardized death, DALY, YLD, and YLL rates of CRC for each country.

Generalized linear mixed model (GLMM) with a gamma distribution and log link function, including a random intercept for country, was used to account for repeated measures across countries. Model fit was assessed using likelihood ratio (LR) tests, the Akaike Information Criterion (AIC), and Bayesian Information Criterion (BIC). Results of GLMM models are reported as β coefficients with corresponding 95% confidence intervals. Statistical analyses were conducted using STATA IC version 13.0 (Stata Corp., College Station, TX, USA). The Gini index was calculated using the ineqdeco package. A *p*-value of less than 0.05 was considered statistically significant.

## 3. Results

The overall age-standardized mortality rate across all EU-27 countries has changed from 22.91 (95% UI: 21.44–23.83) to 17.12 per 100,000 (95% UI: 15.52–18.23) from 1990 to 2021. The age-standardized DALY rate has decreased from 503.53 (95% uncertainty interval (UI): 480.39–520.57) in 1990 to 370.57 per 100,000 (95% UI: 344.49–391.88) in 2021. The trend of age-standardized YLL rate showed a similar pattern to the DALY trend; the rate decreased from 484.93 (95% UI: 462.99–500.61) to 348.94 per 100,000 (95% UI: 324.09–368.44). In contrast, age-standardized YLD showed a slight increase from 18.59 (95% UI: 13.93–23.84) to 21.63 per 100,000 (95% UI: 16.06–28.18) between 1990 and 2021.

**Figure 1:**
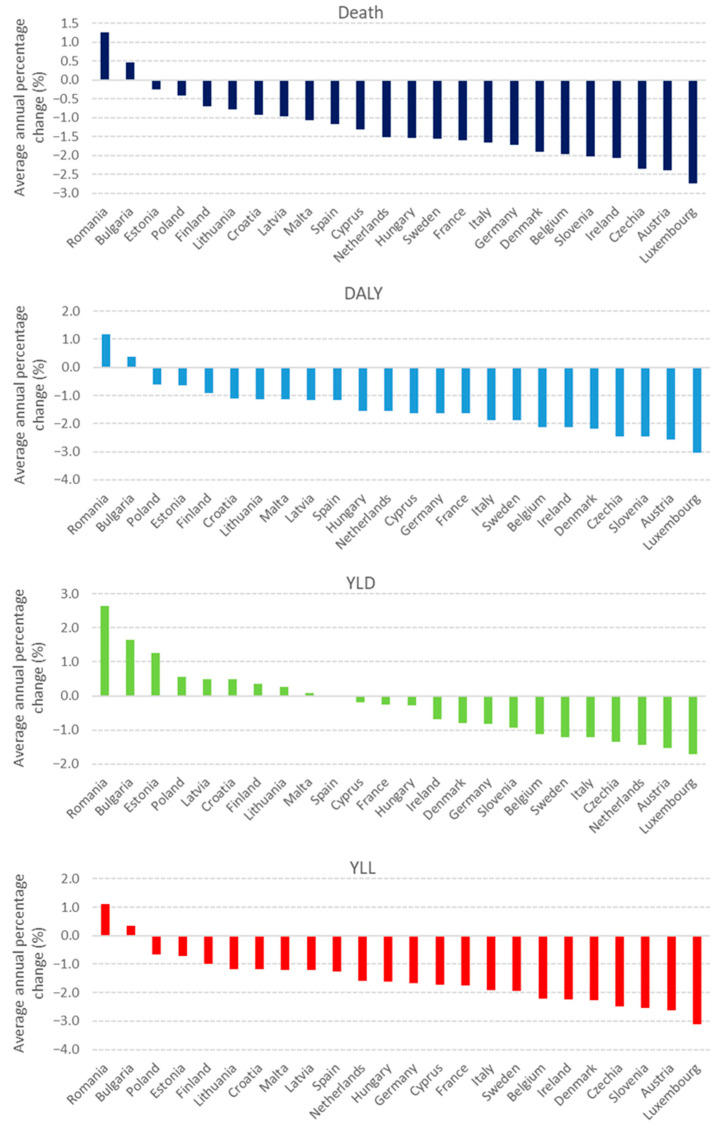
Average annual percent change (AAPC) of age-standardized death, DALY, YLD, and YLL rates in 24 EU member states for the period of 2005–2021.

Figure 1 presents the country-specific average AAPC restricted to the period 2005–2021. The results of the Joinpoint regression indicated that most EU member states experienced significant declines in DALY, mortality rates, and YLL and YLD rates between the study period (2005–2021). Bulgaria and Romania were the only two countries where all four health outcomes increased over the period. The highest improvements were observed in age-standardized YLL rates with average AAPCs in the 1.12% to –3.12% range (Figure 1).

**Figure 2:**
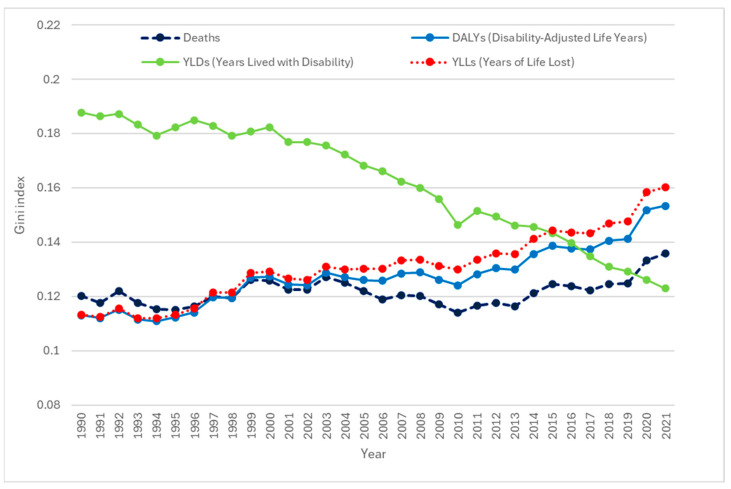
Trends in Gini index of age-standardized death, DALY, YLD, and YLL rates in 24 European Union member states, 1990–2021.

According to the Gini index, health inequality was observed across 24 EU member states (Figure 2). Between 1990 and 2021, the Gini index for age-standardized YLDs in EU member states declined from 0.19 to 0.12. In contrast, inequality in YLL increased from 0.11 to 0.16 over the same period. Inequality for the age-standardized DALY rate showed a similar upward trend, rising from 0.11 in 1990 to 0.15 in 2021. The Gini index for age-standardized death rate increased slightly from 0.12 to 0.14 during the investigated period (Figure 2).

As reported in Table 1, greater number of practicing physicians was related to lower death (β = −0.036, *p* = 0.022), DALY (β = −0.035, *p* = 0.019), YLD (β = −0.060, *p* < 0.001) and YLL (β = −0.034, *p* = 0.022) rates. In contrast, health expenditure in share of GDP and hospital bed availability showed no significant relationship in any model (Table 1).

Among socioeconomic indicators, a higher education attainment was significantly linked to lower disease burden across all four health outcomes (age-standardized death rate: β = −0.009, *p* < 0.001, DALY rates (β = −0.009, *p* < 0.001), YLD rates (β = −0.008, *p* < 0.001) and YLL rates (β = −0.009, *p* < 0.001). Income inequality was positively related to higher death (β = 0.015, *p* = 0.035), DALY (β = 0.019, *p* = 0.005), and YLL (β = 0.019, *p* = 0.004) rates, but not to YLD. The unemployment rate was not a significant factor in any of the models (Table 1).

## 4. Discussion

Our study showed that colorectal cancer burden has decreased across EU member states in recent decades. This improvement is driven mainly by the widespread implementation of organized screening programs, which enable cancers to be detected and treated at an earlier stage of the disease [7]. A recent population-based study of nine European countries reported that screen-detected cancer cases were diagnosed at a more favorable stage than those detected otherwise [25]. Another study found that a 1% rise in screening uptake corresponds to a 2.9% decrease in late-stage incidence rate, which highlights the crucial role of screening in preventing advanced-stage diagnoses [26]. At the same time, advances in treatment, including improved surgical techniques and more effective radiotherapy and chemotherapy regimens, targeted therapies, immunotherapy, and better palliative care, have extended survival and quality of life for CRC patients [27,28,29].

Trend analysis revealed that the improving trend was observed in almost all investigated member states, whereas Bulgaria and Romania were outliers with worsening trends across all burden metrics. In these countries, the unfavorable colorectal cancer outcomes largely stem from the lack of a national, population-based screening program [30], high prevalence of cancer-related risk factors [30,31], and limited access to services due to a shortage of specialist health professionals [30,31].

Country-level differences in CRC burden may reflect variation in healthcare system models, which influence access, equity, and efficiency in cancer prevention and treatment. Additionally, disparities in national policies on CRC screening, health spending priorities, and the strength of primary care infrastructure likely contribute to the observed cross-country heterogeneity [15].

The observed rise in age-standardized YLD reflects the growing population of CRC survivors living with a wide range of long-term physical [32,33,34,35,36] and psychological [35,37] morbidities, which have been described as the survivorship burden in cancer survivors. This trend highlights the need to develop comprehensive survivorship care models focusing on physical rehabilitation, mental health support, and long-term follow-up to improve functional outcomes and quality of life for survivors [35,37].

The inequality analysis revealed that, while disparities in the burden of CRC have decreased in terms of YLD, widening inequalities were observed for mortality, YLL, and DALY over the past decades. These findings indicate that differences in the overall disease burden have not yet been eliminated. The reduction in YLD-related inequality may reflect improvements in equitable access to survivorship care and rehabilitation services across Europe. In contrast, the increasing inequalities in mortality, YLL, and DALYs likely reflect uneven distribution of advancements in early detection, timely diagnosis, and access to effective treatments. Persistent disparities by socioeconomic status remain a major public health challenge in addressing the CRC burden [11].

The observed inverse relationship between physician density and CRC burden is consistent with previous findings that greater availability of physicians (both GPs and specialists) significantly improves CRC outcomes through earlier diagnosis [26,38]. For example, a recent spatial analysis demonstrated that regions with higher primary care physician density experience significantly lower rates of late-stage CRC incidence and mortality [26]. These findings indicate that policy efforts should focus on regions with lower ratios of physicians to population to ensure more equitable access to care.

The strong, consistent link between higher educational attainment and CRC burden suggests that education may serve as a proxy for health literacy, which in turn may influence participation in screening programs [39]. Moreover, low health literacy is not only associated with reduced screening uptake but also linked to a significantly higher incidence of postoperative complications, longer hospital stays, greater hospital charges [40], and poorer quality of life [41] among CRC cancer survivors. Our findings confirm that inadequate education (health literacy) level contributes to the disproportionate burden of CRC faced by socioeconomically disadvantaged populations.

We found a positive link between income inequality and CRC mortality, DALY, and YLL, which reflects that regions with larger income disparities suffer from poorer cancer survival. This finding is consistent with existing literature, including a meta-analysis, which demonstrated that people living in regions with high income inequality exhibit an elevated risk for premature mortality, independent of their socioeconomic status [42]. Moreover, a nationwide Swedish register-based study of colon and rectal cancer patients documented pronounced income-related disparities in life-years lost [43].

The lack of a significant relationship between overall health expenditure as a share of GDP or hospital bed availability and CRC burden highlights the pivotal role of socioeconomic determinants. Recent research revealed that even in regions with high hospital bed density, deprivation-related disparities in CRC detection persist, suggesting that hospital bed availability cannot fully eliminate health disparities due to socioeconomic deprivation [44].

Thus, the major policy implication of this study is that policymakers should invest in expanding access to CRC screening programs and improving early detection in countries with weaker health system performance, particularly those with low healthcare spending and high out-of-pocket costs. Systematic monitoring of CRC screening campaigns is also essential to understand their effectiveness and to inform future optimization efforts [45,46,47]. Additionally, targeted interventions are needed to address CRC disparities among populations with lower socioeconomic status by integrating social protection mechanisms and subsidizing preventive care. Future research is needed to incorporate data on population-based CRC screening programs in EU countries alongside healthcare and socioeconomic indicators. This could provide valuable insights into the effectiveness of national screening campaigns in different settings.

### Strengths and Limitations

One key strength of this study is the use of population-based data to deliver comprehensive estimates of colorectal cancer burden patterns and its relation to healthcare and SES indicators across 24 EU member states. However, several limitations should be acknowledged. First, this ecological study relied on aggregated country-level data, which may hide important subnational heterogeneity in data collection methods, population structure, and lifestyle factors. The health burden may be underestimated because of the hidden morbidity of CRC, particularly in countries with less effective screening programs. Additionally, this study does not account for differences in national healthcare system models (e.g., Bismarckian vs. Beveridge), which may influence the access, delivery, and outcomes of CRC care. Furthermore, the regression analysis was performed for 2005–2021 (due to data availability); extending this period could increase the robustness of the findings. Additional Eurostat indicators with incomplete coverage across countries and years were excluded from the analysis. Despite these limitations, the data sources (Eurostat and GBD) and methodology used in this study were adequate to identify inequalities and trends in CRC burden.

## 5. Conclusions

Our findings suggest that while years lived with disability have become more equitable, reflecting better survivorship care, inequalities in mortality, YLL, and DALYs persist and are widening. Our findings highlight that, alongside expanding organized screening, strengthening physician supply and reducing socioeconomic inequalities are essential strategies for mitigating the unequal burden of colorectal cancer.

## Figures and Tables

**Table 1:** Mixed-effect GLM model for age-standardized death, DALY, YLD, and YLL rates in 24 EU member states.

	Death		DALY		YLD		YLL	
	β (95% CI)	*p*-Value	β (95% CI)	*p*-Value	β (95% CI)	*p*-Value	β (95% CI)	*p*-Value
Healthcare indicators								
Current healthcare expenditure in share of GDP (%)	–0.004 (–0.012, 0.004)	0.283	–0.005 (–0.013, 0.003)	0.195	–0.007 (–0.016, 0.002)	0.132	–0.005 (–0.012, 0.003)	0.224
Practicing physicians (per 1000)	–0.036 (–0.066, –0.005)	**0.022**	–0.035 (–0.064, –0.006)	**0.019**	–0.060 (–0.094, –0.026)	**<0.001**	–0.034 (–0.063, –0.005)	**0.022**
Hospital bed (per 1000)	–0.009 (–0.024, 0.005)	0.193	–0.002 (–0.016, 0.011)	0.762	–0.011 (–0.027, 0.005)	0.186	–0.001 (–0.015, 0.012)	0.847
Socioeconomic indicators								
Proportion of population with tertiary education (%)	–0.009 (–0.012, –0.006)	**<0.001**	–0.009 (–0.012, –0.006)	**<0.001**	–0.008 (–0.012, –0.005)	**<0.001**	–0.009 (–0.012, –0.006)	**<0.001**
Income inequality	0.015 (0.001, 0.029)	**0.035**	0.019 (0.006, 0.032)	**0.005**	0.014 (–0.001, 0.030)	0.068	0.019 (0.006, 0.033)	**0.004**
Unemployment rate (%)	–0.000 (–0.002, 0.002)	0.920	–0.000 (–0.002, 0.002)	0.723	0.000 (–0.002, 0.003)	0.775	–0.000 (–0.002, 0.002)	0.638
Intercept	12.430 (5.785, 19.076)	**<0.001**	17.175 (10.840, 23.509)	**<0.001**	–10.866 (–18.317, –3.415)	**0.004**	18.550 (12.190, 24.911)	**<0.001**
Country intercept	0.038 (0.022, 0.068)	**<0.001**	0.043 (0.024, 0.077)	**<0.001**	0.072 (0.040, 0.129)	**<0.001**	0.042 (0.023, 0.074)	**<0.001**

All models adjusted for year (2005–2021) and included a country-level random intercept. DALY: disability-adjusted life years, YLD: years lived with disability, YLL: years of life lost, CI: confidence interval, GDP: gross domestic product. Significant results are shown in bold.

## Data Availability

Publicly available datasets were analyzed in this study. This data can be found here: http://ghdx.healthdata.org/gbd-results-tool (accessed on 13 May 2025) and https://ec.europa.eu/eurostat/data/database (accessed on 13 May 2025).

## Supplementary Materials

The following supporting information can be downloaded at: https://www.mdpi.com/article/10.3390/cancers17132075/s1, Table S1: Definitions of the socioeconomic and healthcare variables that were used in our study.

## Abbreviations

The following abbreviations are used in this manuscript:

AAPC	average annual percent change
CRC	Colorectal cancer
DALY	Disability-adjusted life years
EU	European Union
GBD	Global Burden of Disease
DALY	Disability-adjusted life years
YLD	Years lived with disability
YLL	Years of life lost
