# Supplementary material for: Socioeconomic and Healthcare Indicators and Colorectal Cancer Burden: Analysis of Eurostat and Global Burden of Disease Study 2021 Data
Source: Cancers (Basel). 2025 Jun 21;17(13):2075. doi: 10.3390/cancers17132075 (PMC12249062; doi:10.3390/cancers17132075)
Supplement: Supplementary file 1 [file cancers-17-02075-s001.zip › cancers-3694012-supplementary.pdf]

Title: Socioeconomic and Healthcare Indicators and Colorectal Cancer Burden: Analysis of Eurostat and Global Burden of Disease Study 2021 Data

Authors: Nóra Kovács and Orsolya Varga

Supplementary file 1: Definitions of the socioeconomic and healthcare variables that were used in our study

**Indicator**	**Code in Eurostat database**	**Definition**
**Healthcare Indicators**		
Current healthcare expenditure in percentage of gross domestic product (GDP)	*hlth_sha11_hc*	The indicator quantifies the economic resources dedicated to all health functions, excluding capital investment. It is expressed in percentage of GDP.
Number of hospital bed	*hlth_rs_bds1*	The indicator refers to the total number of hospital beds available in healthcare institutions (hospitals) and expressed in per 1,000 inhabitants
Practising physicians per 100 000	*hlth_rs_prs2*	The indicator refers the number of physicians available for providing health care services, expressed in per 1,000 population. It serves as a measure of medical workforce availability.
**Socioeconomic Indicators**		
Income quintile share ratio S80/S20 for disposable income	*ilc_di11*	This indicator measures income inequality by comparing the total income received by the top 20% of the population (highest income quintile) to that received by the bottom 20% (lowest income quintile). A higher ratio indicates greater income inequality. The data is sourced from EU-SILC survey.
Unemployment rate	*lfsa_urgan*	This indicator represents the percentage of the population aged 15-64 that is unemployed. The indicator is based on the results of the Euro-pean Labour Force Survey (EU-LFS).
Proportion of population with tertiary education	*edat_lfse_03*	The indicator refers to the population aged 15-74 with tertiary education (ISCED levels 5–8).
